# Asian Ethnicity and Femoral Geometry in Atypical Femur Fractures: Independent or Interdependent Risk Factors?

**DOI:** 10.1002/jbm4.10607

**Published:** 2022-02-17

**Authors:** Nitesh D Dhanekula, Gareth Crouch, Karen Byth, Sue Lynn Lau, Albert Kim, Edward Graham, Andrew Ellis, Roderick J Clifton‐Bligh, Christian M Girgis

**Affiliations:** ^1^ Faculty of Medicine and Health University of Sydney Sydney Australia; ^2^ Department of Orthopaedic Surgery Westmead Hospital Westmead Australia; ^3^ Western Sydney Local Health District (WSLHD) Research and Education Network Westmead Hospital Westmead Australia; ^4^ Department of Diabetes and Endocrinology Westmead Hospital Westmead Australia; ^5^ Department of Endocrinology and Diabetes Royal North Shore Hospital St Leonards Australia; ^6^ Department of Orthopaedic Surgery Royal North Shore Hospital St Leonards Australia; ^7^ Kolling Institute of Medical Research Sydney Australia; ^8^ The Westmead Institute for Medical Research Westmead Australia

**Keywords:** ASIAN ETHNICITY, ATYPICAL FEMUR FRACTURE, BISPHOSPHONATES, FEMORAL GEOMETRY, OSTEOPOROSIS

## Abstract

The earliest reports of atypical femur fractures (AFF) emerged from Asia. In the West, epidemiologic studies report a greater incidence of AFFs among subjects of Asian background. Asian ethnicity is an established risk factor for AFF, but clear mechanisms to explain this risk and implications for the general development of AFF are open questions. Ethno‐specific differences in bisphosphonate action and femoral geometry have been proposed as hypotheses. In a retrospective cohort of 163 female patients presenting with AFFs or typical femur fractures (TFF), relative contributions of Asian ethnicity, proximal femoral geometry, and bisphosphonate use in AFF status were examined. There was a fourfold higher proportion of Asian subjects in the AFF compared with TFF groups (31.6%, 30/95 versus 7.4%, 5/68). Asian subjects had smaller femurs in femoral head, neck, and axial dimensions. A multiple logistic regression model for AFF status was fitted adding Asian ethnicity to three previously reported independent predictors of AFF including femoral geometry, which together comprise the Sydney AFF Score (age ≤80 years, femoral neck width <37 mm than non‐Asian, lateral cortical width at lesser trochanter ≥5 mm). Asian ethnicity was a robust independent predictor of AFF, imparting sevenfold increase in the odds of AFF after adjusting for all three variables (95% confidence interval [CI] 2.2–23.2, *p* = 0.001) or for overall AFF score (95% CI 2.2–22.3 *p* = 0.001). Overall Asian subjects had higher rates of bisphosphonate use than non‐Asian subjects (67.6% versus 47.2%, *p* = 0.034). Among AFF bisphosphonate users, Asian subjects had lower AFF scores than non‐Asians (Sydney AFF Score ≤1, 45.5% Asian subjects versus 22.2% non‐Asian subjects, *p* = 0.05). Asian ethnicity is a strong independent risk factor for AFF, unaccounted for by ethno‐specific differences in proximal femoral geometry. Bisphosphonate use may be associated with a greater predisposition for AFF in Asian subjects compared with non‐Asian subjects. © 2022 The Authors. *JBMR Plus* published by Wiley Periodicals LLC on behalf of American Society for Bone and Mineral Research.

## 1 Introduction

The earliest reports of atypical femur fractures (AFF) emerged from Asia.^(^ ^1^, ^2^ ^)^ AFF, defined as cortical insufficiency fractures occurring in the subtrochanteric femur, have been extensively described in individuals who had been treated with long‐term bisphosphonate use in Singapore,^(^ ^3^, ^4^ ^)^ followed by large case series from Korea^(^ ^5^ ^)^ and Japan.^(^ ^6^ ^)^ In the West, epidemiologic studies report an overrepresentation of Asian subjects among AFF cohorts, with proportions reaching as high as 50%.^(^ ^7^, ^8^ ^)^ In recent studies from Australia and North America, Asian ethnicity conferred a fourfold greater risk of AFF even after adjustment for bisphosphonate use with the highest incidence in people from South‐East Asian countries.^(^ ^9^, ^10^ ^)^

Causes for the greater incidence of AFF among individuals of Asian ethnicity are unclear. Hypotheses include genetic differences in bone turnover and antiresorptive drug metabolism, a tendency to femoral bowing in Asians, resulting in focal accumulation of cortical stress and use of White bone density reference data to determine osteoporosis treatment initiation in Asian subjects.^(^ ^11^, ^12^, ^13^ ^)^

In one study, greater bowing of the femur accounted for more proximally located AFFs in patients of Asian ethnicity.^(^ ^3^ ^)^ A more recent study from Japan suggested that although AFFs in the diaphyseal femur may be related to geometry, overt suppression of bone turnover on histomorphometry characterized subtrochanteric AFFs in people of Asian ethnicity.^(^ ^6^ ^)^ Greater duration of bisphosphonate use among Asian subjects was associated with a higher incidence of AFF in a North American insurance database cohort.^(^ ^7^ ^)^

The elucidation of ethno‐specific determinants of AFF is critical for several reasons. By 2050, more than 60% of the world's hip fractures are projected to occur in Asia and Latin America.^(^ ^14^, ^15^, ^16^ ^)^ A rapid rise in hip fracture across Asian countries has been reported with the incidence predicted to double between 2018 and 2050, at an annual cost exceeding USD 10 billion.^(^ ^15^, ^16^ ^)^

At a global level, a better understanding of ethnic factors leading to AFF may help to clarify the public health message on the safe use of osteoporosis therapies and thereby prevent an avoidable tide of typical hip fractures in Asia over the next three decades. On an individual basis, osteoporosis treatment decisions could be personalized on the basis of “baseline femur morphology,” informed by a clear picture of mechanical factors leading to AFF predominating in Asian subjects. On a molecular and genetic basis, whole‐genome sequencing to establish pathogenic variants predisposing to AFF would be better informed by an understanding of ethnic determinants and racial clusters.^(^ ^17^ ^)^

In this study, we examined the incidence of Asian ethnicity in a cohort of females with atypical and typical subtrochanteric femur fractures from two large institutions in Sydney, Australia. We examined the contribution of Asian ethnicity to the development of AFF, after adjusting for femoral geometric features and rates of bisphosphonate use. We further evaluated the diagnostic value of including Asian ethnicity in the Sydney AFF Score, an algorithm that accurately identifies AFFs among femur fractures using categorical, quantifiable parameters.^(^ ^18^ ^)^ We sought to answer the question as to whether the higher proportion of AFFs in subjects of Asian ethnicity could be attributed to femur geometric indices characteristic of AFFs.

## 2 Materials and Methods

### 2.1 Study population

This multicenter study was conducted at two large institutions in Sydney, Australia, as previously described (Royal North Shore and Westmead Hospitals, ethics approval LNR/17/HAWKE/131).^(^ ^18^ ^)^ Together, these institutions serve a catchment population of 1.6 million people, approximately 7% of the Australian population. A proportion of patients served by these hospitals are from culturally and linguistically diverse backgrounds (40% born outside of Australia, 16.5% born in East Asian countries).^(^ ^19^ ^)^

### 2.2 Atypical femur fracture identification

**Fig. 1:**
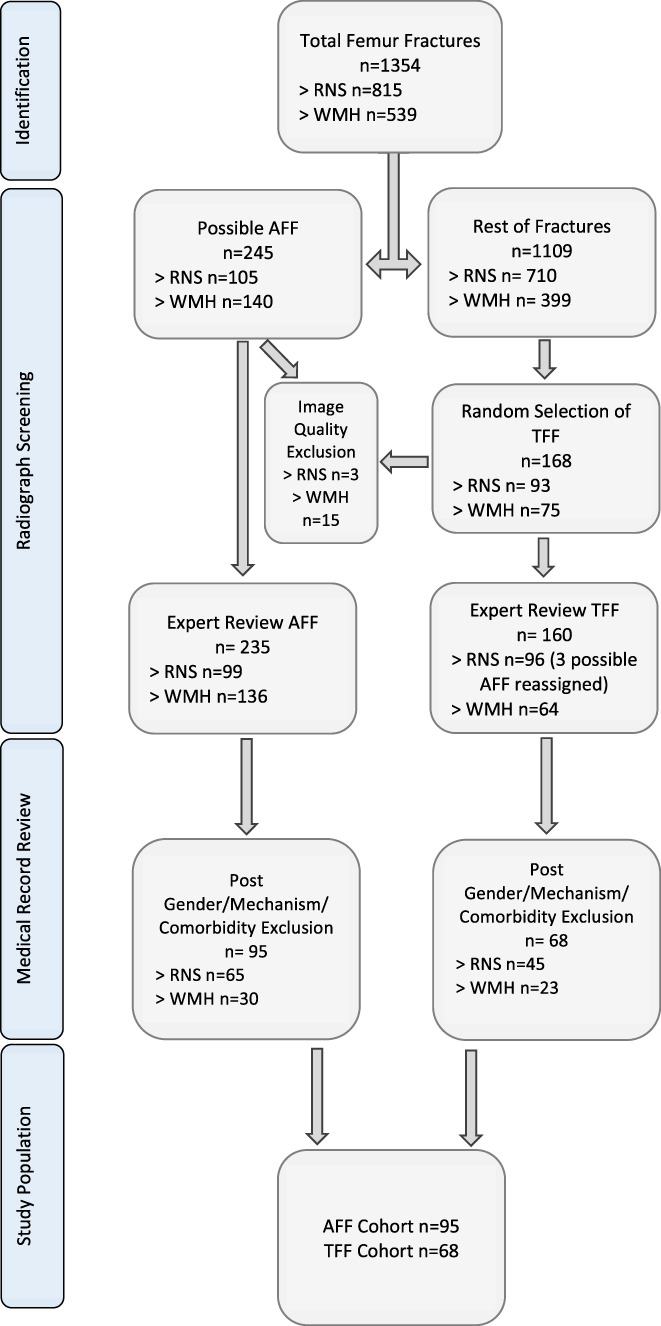
Fracture numbers at each site and stages of exclusion. RNSH = Royal North Shore Hospital; WMH = Westmead Hospital; AFF = atypical femur fractures; TFF = typical femur fractures.

AFFs were identified systematically at both institutions over the 9‐year study period, as described.^(^ ^18^ ^)^ In brief, after identification of intertrochanteric, subtrochanteric, and diaphyseal femoral fractures on the basis of admission and radiology coding, anteroposterior pelvis radiographs were screened for the presence of atypical features based on ASBMR task force criteria for AFFs.^(^ ^18^ ^)^ Femur fractures with atypical features were extracted and a control set of fractures without atypical features (typical femur fractures [TFFs]) were randomly selected. A panel of three expert adjudicators at each site then viewed these X‐rays independently. A final set of AFFs and TFFs was collected, based on the consensus of at least two reviewers at each site. For consistency in femoral geometry evaluation, only female patients were included. Fractures from high trauma, Paget's disease, or bony metastases were excluded. In our previous work, we developed a clinical algorithm, known as the Sydney AFF Score, which accurately identifies AFFs among subtrochanteric femur fractures.^(^ ^18^ ^)^ In this earlier work, fractures from one of the study sites (Royal North Shore Hospital [RNSH]) was used as the discovery set for this score and fractures from the other site (Westmead Hospital) were used for score validation. In the current study, fractures from both sites were pooled into a single cohort to assess the association between ethnicity and AFF status and shown in Fig. 1.

### 2.3 Measurement of proximal femoral geometry

**Fig. 2:**
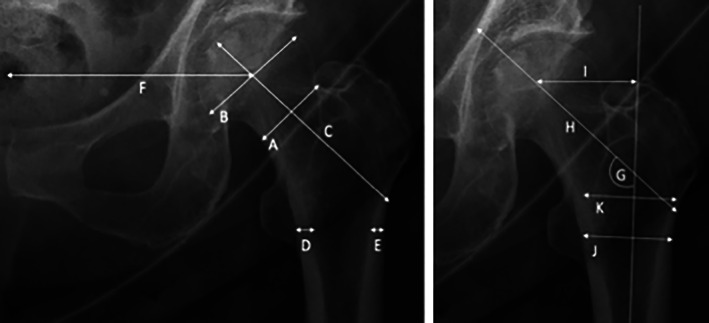
Geometric hip measurements. A = femoral neck width; B = femoral head diameter; C = femoral neck axis length; D = medial cortical width (inferior trochanter); E = lateral cortical width (inferior trochanter); F = distance between femoral head's rotation center and pelvic center; G = neck shaft angle; H = hip axis length; I = femoral offset; J = thickness of femoral shaft at inferior trochanter; K = medullary width 20 mm above lesser trochanter. See Mahjoub and colleagues.^(^ ^20^ ^)^

Measurements were made using the Picture Archiving and Communication System (PACS) electronic medical record (eMR) tool on anteroposterior pelvis radiographs. Measurements relating to femur size and angulation were undertaken as depicted in Fig. 2.

### 2.4 Ethnicity and demographic factors

Review of eMR records was undertaken to collect data on age, medication use, country of birth, and language spoken at home. For the purpose of this study, country of birth (COB) was used as a surrogate marker of ethnicity and dichotomized into two broad groups: Asian and non‐Asian. Asian countries were classified according to Australian Standard Classification of Cultural and Ethnic Groups (ASCCEG) 2019.^(^ ^21^ ^)^ Patients born in a country listed under the South‐East Asian, North‐East Asian, and Southern and Central Asian subcategories of ASCCEG were classified as being of Asian ethnicity.

### 2.5 Statistical analysis

IBM SPSS Statistics Version 25 was used to analyze the data (IBM Corp., Armonk, NY, USA). The following patient attributes were summarized and compared by Asian ethnicity status: age, country of birth, language spoken, any documented bisphosphonate use, and 11 geometric measurements, comprising seven femoral size variables (neck width, head diameter, neck axis length, distance of head rotation center to pelvic center, width at inferior trochanter, hip axis length, and medulla width 20 mm above inferior trochanter), two cortical width variables (medial cortical width at inferior trochanter and lateral cortical width at inferior trochanter), and two angulation variables (neck shaft angle and femoral offset). Means and standard deviations (mean ± SD) were used to summarize continuous variables and frequencies and percentages were used for categorical variables. Two‐sample *t* tests were used to test for differences between groups in the distribution of continuous variables, and chi‐square or exact permutation tests were used for categorical variables. Two‐tailed tests with a significance level of 5% were used throughout.

Multiple logistic regression analysis was used to assess the effect of Asian ethnicity status on AFF status after adjusting for the independent risk factors that contribute to the Sydney AFF Score.^(^ ^18^ ^)^ Odds ratios (OR) and their 95% confidence intervals (95% CI) were used to quantify the strength of association.

## 3 Results

### 3.1 AFF identification

In total, 1354 femur fractures were identified on the basis of radiology and admission codes at both sites (815 at RNSH and 539 at Westmead Hospital). Of these, 245 fractures (105 at RNSH and 140 at Westmead Hospital) were screened as displaying atypical features (NDD, GC). As control, 168 fractures without atypical features were randomly selected (typical femur fractures). A final group of 95 female subjects with an AFF and 68 with a TFF were analyzed following the application of exclusion criteria and adjudication of the fractures by three experts at each site. See Fig. 1 for further information.

### 3.2 Ethnicity and fracture status

Of the 163 subjects in this study, 35 were born in Asia (21.5%), a similar proportion to that in the total population served by the participating institutions. A further 77 subjects (47.2%) were born in Australia, 33 (20.3%) in Europe, and 12 (7.4%) in the Middle East.

The proportion of subjects with Asian ethnicity derived from country of birth was fourfold higher in the AFF group compared with the TFF group (31.6%, 30/95 versus 7.4%, 5/68, OR = 7.2 with 95% CI 2.2–23.2) (see Table 1). Of the 35 Asian‐born subjects in the study, 30 (86%) had an AFF and 5 (14%) had a TFF, whereas subjects born in non‐Asian countries were almost equally divided between the AFF and TFF groups (65 and 63 subjects, respectively). Importantly, selection of the TFF study group from all TFF presentations was random and blinded for ethnicity.

**Table 1:** Fracture Numbers by Country of Origin

	Non‐Asian, *n* (%)	Asian, *n* (%)
Atypical femur fracture	65 (68%)	30 (32%)
Typical femur fracture	63 (93%)	5 (7%)

Asian subjects with an AFF were predominantly of South‐East Asian background (45%) followed by North‐East Asian, and Southern and Central Asian subcategories of ASCCEG (39%, 15% respectively).

### 3.3 Age

Overall, Asian subjects were on average approximately 5 years younger at the time of any femur fracture compared with non‐Asian subjects (75.6 ± 10.2 years versus 80.1 ± 9.9 years, *p* = 0.010) (Table 2). However, there was no significant difference in age between Asian and non‐Asian subjects within the AFF group or in the TFF group (74.9 ± 10.8 versus 76.8 ± 9.7 years, *p* = 0.374, and 80.3 ± 3.7 versus 83.5 ± 6.9 years, *p* = 0.118, respectively). In our original work, an age threshold <80 years was independently associated with AFF versus TFF status. The proportion of subjects aged <80 years did not differ between Asians and non‐Asians, in either the AFF or the TFF group (*p* = 0.446 and *p* = 0.602, respectively). Thus, age differences could not explain the higher proportion of those with Asian ethnicity in the AFF group.

**Table 2:** Summary of Characteristics for All Fractures by Country of Origin

Variable		Non‐Asian (*n* = 128)		Asian (*n* = 35)		*p* Value
		Mean	SD	Mean	SD	
Demographic	Age (years)	80.1	9.9	75.6	10.2	0.010
Femoral size	Neck width	36.63	3.64	34.40	3.05	<0.001
	Head diameter	52.48	4.06	49.92	3.89	<0.001
	Width at lesser trochanter	38.06	4.12	35.45	4.48	0.001
Axial length	Femoral neck axis	107.99	9.43	101.62	10.41	<0.001
	Hip axis	122.97	11.53	115.68	11.14	0.003
	Femoral head rotation center to pelvic center	103.20	9.04	100.44	7.82	0.085
Bone compartment	Medial cortical width at lesser trochanter	6.41	1.94	6.02	1.42	0.365
	Lateral cortical width at lesser trochanter	5.06	1.41	5.32	1.93	0.809
	Medulla width 20 mm above lesser trochanter	38.11	8.01	36.55	8.00	0.317
	Medulla width 50 mm below lesser trochanter	17.63	3.73	14.86	3.41	<0.001
Femur angulation	Femoral offset	40.34	9.48	35.17	6.82	0.001
	Neck shaft angle	130.12	9.00	132.03	8.96	0.119

### 3.4 Measures of femur size

Overall, Asian subjects had significantly smaller femur size (Table 2). Asian subjects had significantly smaller femoral head diameter (49.92 ± 3.89 versus 52.48 ± 4.06 mm, *p* < 0.001), femoral neck width (34.40 ± 3.05 versus 36.63 ± 3.64 mm, *p* < 0.001), and femoral shaft thickness (35.45 ± 4.48 versus 38.06 ± 4.12 mm, *p* = 0.001) compared with non‐Asians. Axial lengths of the femoral neck and hip were also smaller (101.62 ± 10.41 versus 107.99 ± 9.43 mm *p* < 0.001, 115.68 ± 11.14 versus 122.97 ± 11.53 mm, respectively, *p* = 0.003). Measures of specific bone compartments in the femur, however, did not differ by ethnicity, including the lateral cortical width at inferior trochanter (5.32 ± 1.93 versus 5.06 ± 1.41, *p* = 0.809) and medulla width at 20 mm above inferior trochanter (36.55 ± 8 versus 38.11 ± 8.01, *p* = 0.317). Medulla width 50 mm below the inferior trochanter was found to be significantly smaller in Asian subjects (14.86 ± 3.41 versus 17.63 ± 3.73, *p* < 0.001).

Femur size differences by ethnicity were also demonstrated within individual groups. In the AFF group, Asian subjects had femurs that were smaller than non‐Asian subjects (femoral neck width 1.47 mm smaller in Asian subjects, *p* = 0.007; see Table 3 for other measures). Lateral cortical width at inferior trochanter was comparable between Asian and non‐Asian subjects with an AFF (*p* = 0.501). Thus, differences in femur size and cortical width by ethnicity status in the AFF group were consistent with those observed in the fracture cohort at large, as shown in Tables 2 and 3.

**Table 3:** Atypical Femur Fractures by Country of Origin

Variable		Non‐Asian (*n* = 65)		Asian (*n* = 30)		*p* Value
		Mean	SD	Mean	SD	
Demographic	Age (years)	76.8	9.7	74.9	10.8	0.374
Femoral size	Neck width	35.46	2.84	33.99	2.87	0.007
	Head diameter	51.47	3.91	49.75	4.04	0.023
	Width at lesser trochanter	37.20	3.46	35.32	4.49	0.012
Axial length	Femoral neck axis	106.49	8.83	101.50	11.12	0.009
	Hip axis	120.72	10.81	115.18	11.66	0.047
	Femoral head rotation center to pelvic center	101.70	8.80	99.65	7.69	0.168
Bone compartment	Medial cortical width at lesser trochanter	6.62	1.82	6.04	1.50	0.238
	Lateral cortical width at lesser trochanter	5.53	1.29	5.52	1.98	0.501
	Medulla width 20 mm above lesser trochanter	37.42	7.95	36.16	8.04	0.381
	Medulla width 50 mm below lesser trochanter	16.53	3.79	14.54	3.35	0.006
Femur angulation	Femoral offset	39.73	8.62	35.18	7.15	0.008
	Neck shaft angle	131.61	8.73	132.06	9.55	0.381

### 3.5 Measures of femur angulation

In both the overall cohort and within the AFF group, femoral offset was significantly lower in Asian subjects (overall: 35.17 ± 6.82 versus 40.34 ± 9.48 degrees, *p* = 0.001; AFF: 35.18 ± 7.15 vs 39.73 ± 8.62 *p* = 0.008). Neck shaft angle was no different in those of Asian ethnicity both within the overall cohort and specifically amongst those with AFFs (overall 132.03 ± 8.96 versus 130.12 ± 9.00 degrees, *p* = 0.119; AFF 132.06 ± 9.55 vs 131.61 ± 8.73 degrees, *p* = 0.381) (Tables 2 and 3).

### 3.6 Asian ethnicity and Sydney AFF Score

As previously described, the Sydney AFF Score is a tool that accurately identifies AFF among femur fractures using quantitative methods (73.3% sensitivity and 69.6% specificity for AFF).^(^ ^18^ ^)^ This tool was developed using multiple logistic regression and decision tree analyses in a discovery set of fractures from RNSH and independently validated in a separate set of fractures from Westmead Hospital. In the current study, we pooled the discovery and validation sets to examine whether the preponderance of AFFs among subjects of Asian ethnicity could be attributed to components of the Sydney AFF Score.

This was not the case. Asian ethnicity was an independent predictor of AFF versus TFF (OR = 7.2) after adjusting for the three independent components of the score, namely age ≤80 years (OR = 4.5), femoral neck width <37 mm (OR = 4.6), and lateral cortical width ≥5 (OR = 5.4) (Table 4).

**Table 4:** Logistic Regression Model Including Components of the Sydney AFF Score and Asian Country of Birth

	B	SE	Odds Ratio	95% CI		*p* Value
				Lower	Upper	
Individual score components with Asian ethnicity						
Age ≤80 years	1.504	0.414	**4.501**	1.998	10.142	0.000
Femoral neck width <37	1.524	0.419	**4.591**	2.020	10.436	0.000
Lateral cortical width at lesser trochanter ≥5	1.688	0.419	**5.406**	2.377	12.297	0.000
Asian country of origin	1.970	0.600	**7.168**	2.211	23.235	0.001
Constant	−2.434	0.488	0.088			0.000
Sydney AFF Score as a whole with Asian ethnicity						
Score (0–3) per unit increase	1.570	0.277	**4.807**	2.792	8.277	0.000
Asian country of origin	1.938	0.594	**6.947**	2.167	22.263	0.001
Constant	−2.429	0.483	0.088			0.000

Bold indicates significance value (*p* < 0.005).

After accounting for a subject's score (0–3), Asian ethnicity conferred an additional 6.9‐fold increase in the odds of AFF (95% CI 2.2–22. 3, *p* < 0.005) (Table 4).

Asian ethnicity was therefore a robust, independent predictor for AFF status in this cohort and not interdependent on the age and geometric variables that comprise the Sydney AFF Score.

### 3.7 Bisphosphonate use

Bisphosphonate use was significantly higher in patients of Asian ethnicity compared with non‐Asian subjects (67.6% versus 47.2% *p* = 0.034). In AFF subjects, however, there was a similar proportion of bisphosphonate use between Asian and non‐Asian subjects (73.3% versus 71.9%, respectively, *p* = 0.883) (Table 5).

**Table 5:** Frequency of Bisphosphonate Use by Country of Birth and Fracture Type

	Bisphosphonate use	Non‐Asian		Asian		*p* Value
		*n*	%	*n*	%	
Overall Fractures	No	67	52.8%	11	32.4%	0.034
	Yes	60	47.2%	23	67.6%	
Atypical Femur Fractures	No	18	28.1%	8	26.7%	0.883
	Yes	46	71.9%	22	73.3%	
Typical Femur Fractures	No	49	77.8%	3	75.0%	1.000
	Yes	14	22.2%	1	25.0%	

*Note*: Bisphosphonate use unknown for 1 non‐Asian atypical femur fracture and 1 Asian typical femur fracture.

Of the 68 subjects who experienced an AFF with any documented bisphosphonate use, the proportion of patients with a Sydney AFF Score ≤1 was higher in those of Asian versus non‐Asian ethnicity (10/22 45.5% versus 10/45 22.2%, *p* = 0.051). This suggests that greater bisphosphonate use in Asians in the absence of other established risk factors might account for their observed overrepresentation in the AFF group compared with the TFF group.

## 4 Discussion

Bisphosphonates reduce the incidence of minimal trauma fractures by up to 70% and are a central component of the osteoporosis therapeutic armamentarium.^(^ ^22^ ^)^ However, particular individuals are predisposed to the development of AFF in the setting of long‐term bisphosphonate use.^(^ ^23^, ^24^, ^25^ ^)^ At 3 years of treatment, the risk–benefit analysis is strongly in favor of bisphosphonates with the prevention of more than 1200 fragility fractures for every AFF caused.^(^ ^26^, ^27^, ^28^ ^)^ Beyond 5 years, the risk of AFF doubles for every 2‐year period of bisphosphonate use, reaching 100‐fold greater risk compared with bisphosphonate non‐users.^(^ ^29^, ^30^ ^)^ This risk then drops rapidly after a 2‐year bisphosphonate drug holiday, suggesting that predisposed individuals might “reset” their AFF risk while maintaining the benefit of a prolonged antiresorptive response with a well‐timed drug holiday.^(^ ^9^, ^27^, ^29^, ^30^ ^)^ Such decisions on the timing of a bisphosphonate drug holiday rely on a personalized approach, informed by a greater understanding of personal characteristics and risks predisposing to AFF.

Asian ethnicity is a well‐reported risk factor for AFFs. Early reports emerged in Asia,^(^ ^1^, ^2^, ^4^ ^)^ and in AFF cohorts in Western countries, people of Asian ethnicity are overrepresented.^(^ ^7^, ^8^, ^9^, ^10^ ^)^ Precise reasons for this predisposition are unclear. Differences in femoral size and geometry have been hypothesized as potential mechanisms for the development of AFF through the greater concentration of bisphosphonate in smaller femurs and the propagation of local stress and microfracture development in bowed femurs.^(^ ^7^ ^)^ Whether Asian ethnicity per se is a risk factor for AFF or interdependent differences in femur geometry and size remains an open question. This may shed light on the etiology of AFF in general and personalized treatment decisions in people of both Asian and non‐Asian ethnicity.

In our cohort of subjects with subtrochanteric fractures, the proportion of Asian subjects reflected the background population. However, the majority of Asian subjects in our study had an AFF (86%), and Asian subjects had sevenfold greater odds of an AFF compared with a TFF. A recent study reported a similarly higher odds of AFFs among Asian subjects in an Australian population.^(^ ^10^ ^)^ Our study further examined whether femoral geometric differences and the combination of patient factors known collectively as the Sydney AFF Score^(^ ^18^ ^)^ might account for this.

Asian subjects had smaller femurs, in femoral head, neck, and axial length dimensions, compared with non‐Asians in the study cohort at large and separately within AFF and TFF groups. Femoral size differences did not specifically account for the higher incidence of Asian subjects in the AFF group. Similarly, measures of femur angulation were consistent across the groups and did not differ by fracture type. Femoral offset was significantly lower in Asian subjects with no reported difference in neck shaft angle between those of Asian versus non‐Asian ethnicity. The femur varus angle theory proposes that greater femur angulation in Asian subjects might predispose to greater stress accumulation and AFF development.^(^ ^11^, ^12^ ^)^ In a large group of femurs examined by 3D CT, radius of curvature (ROC) was reduced in subjects of Asian ethnicity, indicating a greater degree of femoral bowing.^(^ ^13^ ^)^ After adjustment for femur length, ROC was not significantly different across Asian and non‐Asian subjects,^(^ ^13^ ^)^ suggesting an association between smaller femurs and greater femoral bowing in Asian subjects.

Asian subjects in our study were approximately 5 years younger than non‐Asians. However, Asians were found to have a comparable age within each fracture group. The proportion of subjects within each fracture type who were <80 years, an age threshold that was independently associated with AFF status,^(^ ^18^ ^)^ did not differ by ethnicity. Hence, age differences did not account for the higher proportion of Asian subjects in the AFF group.

A multiple logistic regression model for AFF status was fitted adding Asian ethnicity to components of the Sydney AFF Score. This previously published score combines three independent variables to accurately identify subjects with an AFF among subtrochanteric femur fractures: age ≤80 years, femoral neck width <37 mm, and lateral cortical width at lesser trochanter ≥5 mm.^(^ ^18^ ^)^ Asian ethnicity remained a robust independent predictor of AFF status, imparting a sevenfold increase in the odds of AFF after adjusting for all three component score variables, or a 6.9‐fold increase in the odds of AFF adjusted for the overall score. Thus, cortical width, femur size and age, either in isolation or as a composite of AFF identification, could not account for the higher proportion of Asian subjects among those with AFF. Ethnicity and score components were independent determinants of AFF status in this group of femur fractures.

A significantly higher rate of bisphosphonate use was recorded in Asian subjects. Among AFF subjects with a score of ≤1, Asians had a higher rate of bisphosphonate use compared with non‐Asians. This raises the possibility that bisphosphonate use may preferentially heighten the risk of AFF in Asian versus non‐Asian subjects in the absence of multiple other risk determinants for AFF. Higher prescription rates among Asian subjects, possibly on the basis of overdiagnosis using White bone density reference intervals, may thereby predispose this ethnic group to AFF. To further examine this important question, larger‐scale studies are required, examining prescription databases, clinical and bone density parameters, and fracture outcomes among ethnicities.

Strengths of this study include meticulous review of 9 years of femur fractures at two large tertiary referral centers (2008–2017), including expert adjudication and review of eMR records to identify the AFF group, followed by careful subgroup analysis by ethnicity. This study comprises the largest AFF group in Australia and is comparable to other groups from Scandinavia and North America. This is the first work to quantify and compare measurements of femur size, angulation, and composite AFF score prediction on the basis of ethnicity, attempting to shed light on the significantly higher risk of AFF amongz people of Asian ethnicity.

This study has limitations. Results are based solely on recorded country of birth. Whether children of Asian migrants and subsequent generations of people of Asian heritage might retain skeletal characteristics of their parents' country is unclear. Age of migration is another potential confounder. For various reasons, Asian subjects in our study (mean age 75.6 years) were very likely to have migrated during adulthood and after the mid‐1970s.^(^ ^31^ ^)^ South‐Asian patients were also included among our predominantly East‐Asian cohort, although femoral characteristics between Asian countries may differ. As noted in our original article, the use of fracture radiographs poses technical challenges in the measurement of femoral geometry because of rotational deformity and altered posture. To circumvent this, the contralateral, unfractured femur was measured. Distal femur geometry was not assessed, and hence the contribution of femoral bowing to the development of AFF in Asian subjects remains an open question. There were a small number of Asian subjects in the TFF group (*n* = 5), an unavoidable limitation given the propensity for AFFs among Asian subjects and the random selection of TFFs sampled for study purposes.

The question of ethno‐specific variations in AFF is key to our understanding of the etiology of this serious complication of bisphosphonates. This is the first study to examine associations between AFF status, Asian ethnicity, and femoral geometry in patients with subtrochanteric femur fractures. Asian ethnicity was a robust, independent predictor of AFF status, unrelated to characteristic differences in femur size and shape. The Sydney AFF Score remained an accurate diagnostic marker for AFF after adjusting for Asian ethnicity. The higher proportion of Asian subjects in the AFF group was not explained by the score or by its three component variables. Thus ethnicity, age, and femoral geometry remained independent and not interdependent determinants of AFF among this group of femur fractures. This raises an important question: If femoral geometry cannot explain the higher proportion of Asian people in the AFF group, then what does? This study does not answer this question but offers a hint. A higher rate of bisphosphonate prescription in Asian subjects whose diagnosis of osteoporosis may be based on White bone density reference intervals is one potential explanation. Differences in pharmacologic and antiresorptive responses of bisphosphonates are possible, with a potentially lower AFF threshold in shorter, lighter individuals of a different ethnic background in whom material properties of bone may differ.^(^ ^32^ ^)^ Decisions on bisphosphonate drug holidays and strategies to individualize the risk–benefit analyses of long‐term bisphosphonate treatment need to be determined—ethnic variations in AFF would be a good place to start in answering this question. Personalized medicine has revolutionized the treatment of cancer. In time, a greater understanding of ethnic, pharmacogenomic, and biomechanical factors may yet shift the therapeutic goalposts in osteoporosis, leading to antiresorptive drug choices and treatment intervals tailored to the individual.

## Disclosures

All authors state that they have no conflicts of interest.

5

### Peer Review

The peer review history for this article is available at https://publons.com/publon/10.1002/jbm4.10607.

## References

[1] Koh JS , Goh SK , Png MA , Kwek EB , Howe TS . Femoral cortical stress lesions in long‐term bisphosphonate therapy: a herald of impending fracture? J Orthop Trauma. 2010;24(2):75‐81. 20101130 10.1097/BOT.0b013e3181b6499b

[2] Kwek EB , Goh SK , Koh JS , Png MA , Howe TS . An emerging pattern of subtrochanteric stress fractures: a long‐term complication of alendronate therapy? Injury. 2008;39:224‐231. 18222447 10.1016/j.injury.2007.08.036

[3] Schilcher J , Howe TS , Png MA , Aspenberg P , Koh JSB . Atypical fractures are mainly subtrochanteric in Singapore and diaphyseal in Sweden: a cross‐sectional study. J Bone Miner Res. 2015;30(11):2127‐2132. 25950861 10.1002/jbmr.2547

[4] Soh HH , Chua ITH , Kwek EBH . Atypical fractures of the femur: effect of anterolateral bowing of the femur on fracture location. Arch Orthop Trauma Surg. 2015;135(11):1485‐1490. 26286640 10.1007/s00402-015-2297-4

[5] Lee YK , Ahn S , Kim KM , Suh CS , Koo KH . Incidence rate of atypical femoral fracture after bisphosphonates treatment in Korea. J Korean Med Sci. 2018;33(5):e38. 29349947 10.3346/jkms.2018.33.e38 PMC5773851

[6] Oh Y , Yamamoto K , Hashimoto J , et al. Biological activity is not suppressed in mid‐shaft stress fracture of the bowed femoral shaft unlike in "typical" atypical subtrochanteric femoral fracture: a proposed theory of atypical femoral fracture subtypes. Bone. 2020;137:115453. 32470545 10.1016/j.bone.2020.115453

[7] Lo JC , Hui RL , Grimsrud CD , et al. The association of race/ethnicity and risk of atypical femur fracture among older women receiving oral bisphosphonate therapy. Bone. 2016;85:142‐147. 26769007 10.1016/j.bone.2016.01.002 PMC5108728

[8] Lo JC , Huang S , Lee GA , et al. Clinical correlates of atypical femoral fracture. Bone. 2012;51(1):181‐184. 22414379 10.1016/j.bone.2012.02.632

[9] Black DM , Geiger EJ , Eastell R , et al. Atypical femur fracture risk versus fragility fracture prevention with bisphosphonates. N Engl J Med. 2020;383(8):743‐753. 32813950 10.1056/NEJMoa1916525 PMC9632334

[10] Nguyen HH , Lakhani A , Shore‐Lorenti C , et al. Asian ethnicity is associated with atypical femur fractures in an Australian population study. Bone. 2020;135:115319. 32179169 10.1016/j.bone.2020.115319

[11] Haider IT , Schneider PS , Edwards WB . The role of lower‐limb geometry in the pathophysiology of atypical femoral fracture. Curr Osteoporos Rep. 2019;17(5):281‐290. 31410718 10.1007/s11914-019-00525-x

[12] Starr J , Tay YKD , Shane E . Current understanding of epidemiology, pathophysiology, and management of atypical femur fractures. Curr Osteoporos Rep. 2018;16(4):519‐529. 29951870 10.1007/s11914-018-0464-6 PMC6061199

[13] Thiesen DM , Berger‐Groch J , Ntalos D , et al. Femoral antecurvation—a 3D CT analysis of 1232 adult femurs. PLoS One. 2018;13(10):e0204961. 30300421 10.1371/journal.pone.0204961 PMC6177158

[14] Cheung CL , Ang SB , Chadha M , et al. An updated hip fracture projection in Asia: the Asian Federation of Osteoporosis Societies study. Osteoporos Sarcopenia. 2018;4(1):16‐21. 30775536 10.1016/j.afos.2018.03.003 PMC6362950

[15] Curtis EM , Moon RJ , Harvey NC , Cooper C . The impact of fragility fracture and approaches to osteoporosis risk assessment worldwide. Bone. 2017;104:29‐38. 28119181 10.1016/j.bone.2017.01.024 PMC5420448

[16] Dhanwal DKM , Dennison EM , Harvey NC , Cooper C . Epidemiology of hip fracture: worldwide geographic variation. Indian J Orthop. 2011;45(1):15‐22. 21221218 10.4103/0019-5413.73656 PMC3004072

[17] Nguyen HH , van de Laarschot DM , Verkerk A , et al. Genetic risk factors for atypical femoral fractures (AFFs): a systematic review. JBMR Plus. 2018;2(1):1‐11. 30283886 10.1002/jbm4.10024 PMC6124156

[18] Crouch G , Dhanekula ND , Byth K , et al. The Sydney AFF Score: a simple tool to distinguish females presenting with atypical femur fractures versus typical femur fractures. J Bone Miner Res. 2021;36(5):910‐920. 33528853 10.1002/jbmr.4255

[19] Australian Bureau of Statistics . Census 2016, Data on Local Government Areas.

[20] Mahjoub Z , Jean S , Leclerc JT , et al. Incidence and characteristics of atypical femoral fractures: clinical and geometrical data. J Bone Miner Res. 2016;31(4):767‐776. 26588590 10.1002/jbmr.2748

[21] Australian Bureau of Statistics. Australian Standard Classification of Cultural and Ethnic Groups . Available at: https://www.abs.gov.au/AUSSTATS/abs@.nsf/DetailsPage/1249

[22] Milat F , Ebeling PR . Osteoporosis treatment: a missed opportunity. Med J Aust. 2016;205(4):185‐190. 27510350 10.5694/mja16.00568

[23] Girgis CM , Seibel MJ . Atypical femur fractures: a complication of prolonged bisphosphonate therapy? Med J Aust. 2010;193(4):196‐198. 20712536 10.5694/j.1326-5377.2010.tb03865.x

[24] Girgis CM , Seibel MJ . Atypical femur fractures: a review of the evidence and its implication to clinical practice. Ther Adv Musculoskelet Dis. 2011;3(6):301‐314. 22870488 10.1177/1759720X11416270 PMC3383496

[25] Girgis CM , Seibel MJ . Guilt by association? Examining the role of bisphosphonate therapy in the development of atypical femur fractures. Bone. 2011;48(5):963‐965. 21349355 10.1016/j.bone.2011.02.013

[26] Black DM , Rosen CJ . Clinical practice. Postmenopausal osteoporosis. N Engl J Med. 2016;374(3):254‐262. 26789873 10.1056/NEJMcp1513724

[27] Schilcher J , Koeppen V , Aspenberg P , Michaelsson K . Risk of atypical femoral fracture during and after bisphosphonate use. Acta Orthop. 2015;86(1):100‐107. 25582459 10.3109/17453674.2015.1004149 PMC4366670

[28] Black DM , Abrahamsen B , Bouxsein ML , Einhorn T , Napoli N . Atypical femur fractures: review of epidemiology, relationship to bisphosphonates, prevention, and clinical management. Endocr Rev. 2019;40(2):333‐368. 30169557 10.1210/er.2018-00001

[29] Lo JC , Grimsrud CD , Ott SM , Chandra M , Hui RL , Ettinger B . Atypical femur fracture incidence in women increases with duration of bisphosphonate exposure. Osteoporos Int. 2019;30(12):2515‐2520. 31555883 10.1007/s00198-019-05112-5 PMC7449240

[30] Schilcher J , Michaëlsson K , Aspenberg P . Bisphosphonate use and atypical fractures of the femoral shaft. N Engl J Med. 2011;364(18):1728‐1737. 21542743 10.1056/NEJMoa1010650

[31] Parliament of Australia . Statement on Asian Immigration. Available at: https://www.aph.gov.au/sitecore/content/Home/About_Parliament/Parliamentary_Departments/Parliamentary_Library/Publications_Archive/CIB/CIB9697/97cib16#DEMOGRAPHIC

[32] Lloyd AA , Gludovatz B , Riedel C , et al. Atypical fracture with long‐term bisphosphonate therapy is associated with altered cortical composition and reduced fracture resistance. Proc Natl Acad Sci U S A. 2017;114(33):8722‐8727. 28760963 10.1073/pnas.1704460114 PMC5565436

